# Synergistic antidepressant-like effect of the joint administration of caffeine and NMDA receptor ligands in the forced swim test in mice

**DOI:** 10.1007/s00702-015-1467-4

**Published:** 2015-10-28

**Authors:** Anna Serefko, Aleksandra Szopa, Aleksandra Wlaź, Sylwia Wośko, Piotr Wlaź, Ewa Poleszak

**Affiliations:** Chair and Department of Applied Pharmacy, Medical University of Lublin, Chodźki 1, 20-093 Lublin, Poland; Department of Pathophysiology, Medical University of Lublin, Lublin, Poland; Department of Animal Physiology, Institute of Biology and Biochemistry, Maria Curie-Skłodowska University, Lublin, Poland

**Keywords:** Caffeine, Antidepressant-like activity, Forced swim test, NMDA receptor ligands, Mice

## Abstract

The optimal treatment of depressed patients remains one of the most important challenges concerning depression. The identification of the best treatment strategies and development of new, safer, and more effective agents are crucial. The glutamatergic system seems to be a promising drug target, and consequently the use of the NMDA receptor ligands, particularly in co-administration with other substances exerting the antidepressant activity, has emerged among the new ideas. The objective of this study was to examine the effect of caffeine on the performance of mice treated with various NMDA modulators in the forced swim test. We demonstrated a significant interaction between caffeine (5 mg/kg) and the following NMDA receptor ligands: MK-801 (an antagonist binding in the ion channel, 0.05 mg/kg), CGP 37849 (an antagonist of the glutamate site, 0.312 mg/kg), L-701,324 (an antagonist of the glycine site, 1 mg/kg), and d-cycloserine (a high-efficacy partial agonist of the glycine site, 2.5 mg/kg), while the interaction between caffeine and the inorganic modulators, i.e., Zn^2+^ (2.5 mg/kg) and Mg^2+^ (10 mg/kg), was not considered as significant. Based on the obtained results, the simultaneous blockage of the adenosine and NMDA receptors may be a promising target in the development of new antidepressants.

## Introduction

According to World Health Organization (WHO), depression is one of the five most serious health problems in the world the prevalence of which continues to increase. It is estimated that by 2020, major depression will be the second most common illness (after cardiovascular disease) and cause of premature death (Murray and Lopez 1997). Patients with mood disorders are at increased risk of cardiac mortality and morbidity (Carney et al. 2002) as well as at increased risk of suicide attempts (Goldney 2003; Lowe et al. 2008; Tondo et al. 2003). Although the medical world realizes that the etiology of depression is complex and it involves numerous biological, psychological, and social factors, there are still several unsolved “mysteries” around this mental disease. Scientific research on epidemiology, risk factors, and development of depression has been continuously performed for decades. One of the most important remaining challenges is the optimal treatment of depressed patients. The therapeutic efficiency of antidepressant medications used in contemporary clinical practice reaches approximately 70 % (Keller and Boland 1998; Solomon et al. 2000). Besides, the available drugs evoke a great number of adverse reactions and it requires at least 2 weeks of administration to observe the first signs of improvement. Thus, the identification of the best treatment strategies and the development of new, safer, and more effective agents are crucial (Skolnick et al. 2009). Scientists focus on both novel chemical compounds with an antidepressant potential and the unique combinations of well-known drugs. Mineral substances (such as magnesium or zinc) and natural herbal extracts from widely recognized herbal materials (such as *Hyperici herba, Panax ginseng, Eleutherococcus senticosus*, *Colae semen*) and from less known ones (like *Ilex paraguariensis*) are in the limelight, as well (Nowak 2009).

Since a growing body of evidence indicates that the glutamatergic system is implicated in the pathogenesis of depression, antagonism of different sites at the NMDA receptor complex has emerged among the new treatment strategies. For example, d-cycloserine, known as a partial agonist of the NMDA-associated glycine site, acts at the higher doses (above 100 mg/day) as a functional antagonist of the NMDA receptor. As a well-tolerated adjuvant medication, it produces a significant antidepressant effect in patients refractory to treatment (Heresco-Levy et al. 2013). Similarly, a nonspecific NMDA receptor antagonist, amantadine, potentiates the effects of conventional antidepressant therapy in nonresponsive patients (Rogóż et al. 2007). Traxoprodil, a GluN2B subunit-selective antagonist, appears to be effective and safe for patients with treatment-refractory major depressive disorder (Preskorn et al. 2008). Several authors have reported the beneficial effects of ketamine and memantine treatment for depression; however, the efficacy of these uncompetitive antagonists of the NMDA receptor complex needs further clinical confirmation (Berman et al. 2000; Chilukuri et al. 2014; Muhonen et al. 2008; Zarate et al. 2006). The cationic antagonists, i.e., zinc and magnesium, improve the symptoms of depression by themselves (Eby et al. 2011; Nowak et al. 2005), and they enhance the efficacy of the antidepressant drugs (Poleszak 2007; Poleszak et al. 2005; Szewczyk et al. 2002).

Despite possessing a significant antidepressant potential, most of the NMDA receptor inhibitors given at pharmacologically active doses induce severe undesirable reactions because of which they cannot be used in the treatment of patients with mood disorders (Farlow 2004; Tricklebank et al. 1989; Willetts et al. 1990). Nevertheless, the glutamatergic system seems to be a promising drug target and consequently, the efforts to minimize the toxicity of the NMDA ligands are undertaken. According to the literature data, the synergistic antidepressant effect was observed after concurrent administration of classical antidepressant drugs, such as fluoxetine, imipramine, citalopram, or reboxetine and the NMDA receptor blockers, when both were given at the sub-therapeutic doses (Poleszak et al. 2005, 2011; Szewczyk et al. 2002). Moreover, the outcomes of our previous experiments revealed that the combination of two NMDA receptor ligands modulating the distinct receptor sites induces more pronounced antidepressant-like effect than monotherapy (Poleszak et al. 2013).

Caffeine is known as the most widely consumed central-nervous-system stimulant that predominantly acts through inhibition of the A_1_- and A_2A_-adenosine receptors (Smith et al. 2006). Surprisingly, caffeine use has been reported to both increase depressive symptoms (Gilliland and Andress 1981; Greden et al. 1978; James and Crosbie 1987) and decrease the risk of clinical depression (Hintikka et al. 2005). Some authors claim that the antidepressant effect of caffeine appears after administration of low doses (Gan et al. 2009), whereas a long-term use of particularly high doses of caffeine produces depressive symptoms in humans (Smith 2002). Interestingly, psychiatric patients have been reported to consume more caffeine in their diet than the healthy ones (Scott et al. 1989). Although the exact molecular mechanism of action has not yet been discovered, it is alleged that caffeine may indirectly impact the neurotransmissions responsible for the pathomechanism of the affective disorders (i.e., noradrenergic, serotoninergic, dopaminergic, and glutamatergic systems) (Smith et al. 2006). In fact, both clinical and preclinical studies have indicated a link between depression-like behavior and the adenosine modulatory system, since the adenosine receptors A_1_ and A_2A_ control the release of serotonin, corticotrophin-cortisol/corticosterone, and glutamate and influence the hypothalamic–pituitary–adrenal axis to some extent (Fredholm et al. 2005). Similarly, the available data regarding adenosine activity are not consistent. Several reports have demonstrated that adenosine and its analogs induce behavioral despair in animal models (El Yacoubi et al. 2003), whereas other authors (Kaster et al. 2004, 2005a, b, 2007, 2012) have shown an antidepressant-like effect in mice in the forced swim test (FST) after acute administration of this neuromodulator. According to Kaster et al. (2004, 2005a, b, 2007, 2012), the observed results depended most probably on the interaction between A_1_, A_2A_, and 5-HT_1A_ receptors, l-arginine-nitric oxide pathway, K^+^ channels as well as on the inhibition of the NMDA receptors.

Since caffeine is a widely consumed psychoactive substance, it seems highly important to evaluate its potential for drug interactions with the existing and investigational treatments. The aim of our study was to assess the influence of caffeine on the performance of mice treated with various NMDA modulators in the FST.

## Materials and methods

### Animals

All experiments were performed with the use of naïve adult male Albino Swiss mice weighing approximately 25–30 g (10–12 weeks old), purchased from a licensed breeder (Kołacz, Warszawa, Poland). The animals were housed and tested in accordance with European Union and Polish legislative acts concerning animal experimentation. Each mouse was tested only once. The behavioral tests started after at least a 1-week period of animals’ acclimation to the laboratory environment. The mice were kept in groups of 10 in standard Makrolon cages (425 mm × 265 mm × 150 mm) on wood shavings under controlled housing conditions (ambient temperature 20–23 °C, relative humidity 45–55 %, 12/12 h light/dark cycle, light on at 6:00 a.m., chow pellets, and tap water continuously available). The experiments were conducted between 8 a.m. and 3 p.m. to minimize circadian influences. The experimental groups consisted of 7–10 animals, randomly assigned. Separate groups of mice were taken for the FST and the spontaneous locomotor activity studies. The Local Ethics Committee at the Medical University of Lublin approved all experimental procedures while all efforts were made to minimize animal suffering and reduce the number of mice used in the experiments.

### Drugs

The following substances were used: caffeine (1,3,7-trimethylxanthine, Sigma-Aldrich, Poznań, Poland), imipramine (30 mg/kg, Polfa, Kraków, Poland), MK-801 (dizocilpine, (5R,10S)-(+)-5-methyl-10,11-dihydro-5H-dibenzo[a,d]cyclohepten-5,10-imine hydrogen maleate, 0.05 mg/kg, Sigma-Aldrich), CGP 37849 (dl-(E)-amino-4-methyl-5-phosphono-3-pentenoic acid, 0.3 mg/kg, Abcam Biochemicals, Cambridge, UK), L-701,324 (7-chloro-4-hydroxy-3-(3-phenoxy)phenylquinolin-2[1H]-one, 1 mg/kg, Sigma-Aldrich), d-cycloserine (d-4-amino-3-isoxazolidone, 2.5 mg/kg, Sigma-Aldrich), magnesium hydroaspartate (Farmapol, Poznań, Poland), and zinc hydroaspartate (Farmapol). Both the active dose of imipramine and the ineffective doses of the NMDA receptor ligands were selected on the basis of the outcomes of our previous experiments (Poleszak et al. 2005, 2013). Doses of magnesium and zinc refer to pure magnesium and zinc ions—10 and 2.5 mg/kg, respectively. Caffeine, imipramine, MK-801, CGP 37849, d-cycloserine, and the salts of divalent cations were dissolved in physiological saline, while L-701,324 was suspended in a 1 % aqueous solution of Tween 80 (POCH, Gliwice, Poland). All the solutions and the suspension were prepared immediately prior to the experiments and they were given intraperitoneally (i.p.). All agents except for caffeine and magnesium hydroaspartate were injected 60 min before behavioral testing, while caffeine and magnesium hydroaspartate were administered 40 and 30 min prior to the experiments, respectively. The control groups received saline. The volume of vehicle or drug solutions/suspension for i.p. administration was 10 ml/kg.

### Forced swim test

The forced swim test was conducted as described in the literature (Porsolt et al. 1977). Each animal was placed in a glass cylinder (height 25 cm, diameter 10 cm) containing 10 cm of water at the temperature of 23–25 °C and left for 6 min. As little immobility was observed during the first 2 min, the total duration of immobility was recorded between the 2nd and the 6th minute of the experiment. The animal was judged to be immobile when it stopped struggling and was making only the movements necessary to keep its head above the water level.

### Spontaneous locomotor activity

The determination of the spontaneous locomotor activity of animals is a complementary procedure which is performed to confirm that the interpretation of the outcomes obtained in the FST is not disturbed by changes in the animals’ locomotion. In our experiments, the spontaneous locomotor activity was measured with Opto-Varimex-4 Auto-Track (Columbus Instruments, Columbus, OH, USA)—an automatic device with four transparent cages covered with movable lids, a set of four infrared emitters (with laser beams), and four detectors monitoring animal movements. Each mouse was placed in an individual cage and left there for 6 min. The spontaneous locomotor activity was evaluated between the 2nd and the 6th minute, which corresponds with the time interval analyzed in the FST. It was measured as a distance (in cm) traveled by a mouse.

### Statistical analysis

The obtained data were assessed by the one-way analysis of variance (ANOVA) followed by Dunnett’s post hoc test or by the two-way analysis of variance (ANOVA) followed by Bonferroni’s post hoc test, depending on the experimental design. All results are presented as the mean ± standard error of the mean (SEM). *p* was considered as statistically significant when *p < 0.05, **p < 0.01, ***p < 0.001. Statistical analysis was performed with GraphPad Prism version 5.03 for Windows (GraphPad Software, San Diego, CA, USA).

## Results

### Effect of an acute administration of caffeine on the FST in mice

**Fig. 1:**
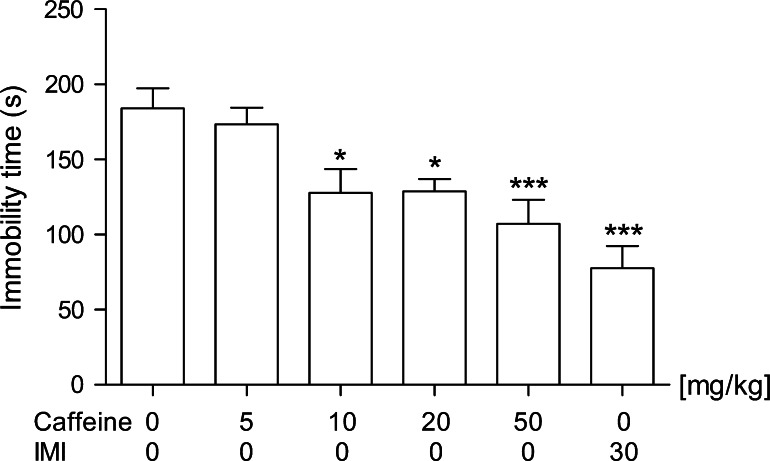
Effect of an acute administration of different caffeine doses on immobility time in the FST in mice. Caffeine (5, 10, 20, or 50 mg/kg) and imipramine (IMI, 30 mg/kg) were administered i.p. 40 and 60 min before the experiment, respectively. The acute administration of imipramine was used as a positive control. The values represent the mean + SEM (*n* = 8–10 mice per group). *p < 0.05, ***p < 0.001 versus saline-treated group (one-way ANOVA followed by Dunnett’s post hoc test)

As shown in Fig. 1, caffeine after i.p. administration exerted an antidepressant-like effect in the FST in mice. The antidepressant potential was significant within the dose range of 10–50 mg/kg. One-way ANOVA revealed the following statistics: *F*(5,48) = 8.900, *p* < 0.0001. The concentration of 5 mg/kg appeared to be a sub-effective dose. Imipramine, used as a positive control at a dose of 30 mg/kg, also considerably reduced the immobility time of mice left in the inescapable situation, which confirmed the correctness of the applied methodology. The tested doses of caffeine did not influence the locomotion of mice (Table 1).

**Table 1:** Effect of an acute administration of caffeine on the spontaneous locomotor activity in mice

Treatment	Dose (mg/kg)	Activity counts between the 2nd and the 6th minute (cm)
Saline	–	956.4 ± 68.87
Caffeine	5	1057 ± 54.20
Caffeine	10	1145 ± 139.8
Caffeine	20	1143 ± 132.1
Caffeine	50	1107 ± 84.90

Data represent the mean ± SEM (*n* = 8 mice per group). Caffeine was administered *i.p.* 40 min before the test at the following doses: 5, 10, 20, or 50 mg/kg. The difference was considered statistically significant if *p* < 0.05 (one-way ANOVA [*F*(4,35) = 0.5921, *p* = 0.670])

### Effect of a joint administration of caffeine and MK-801 on the FST in mice

**Fig. 2:**
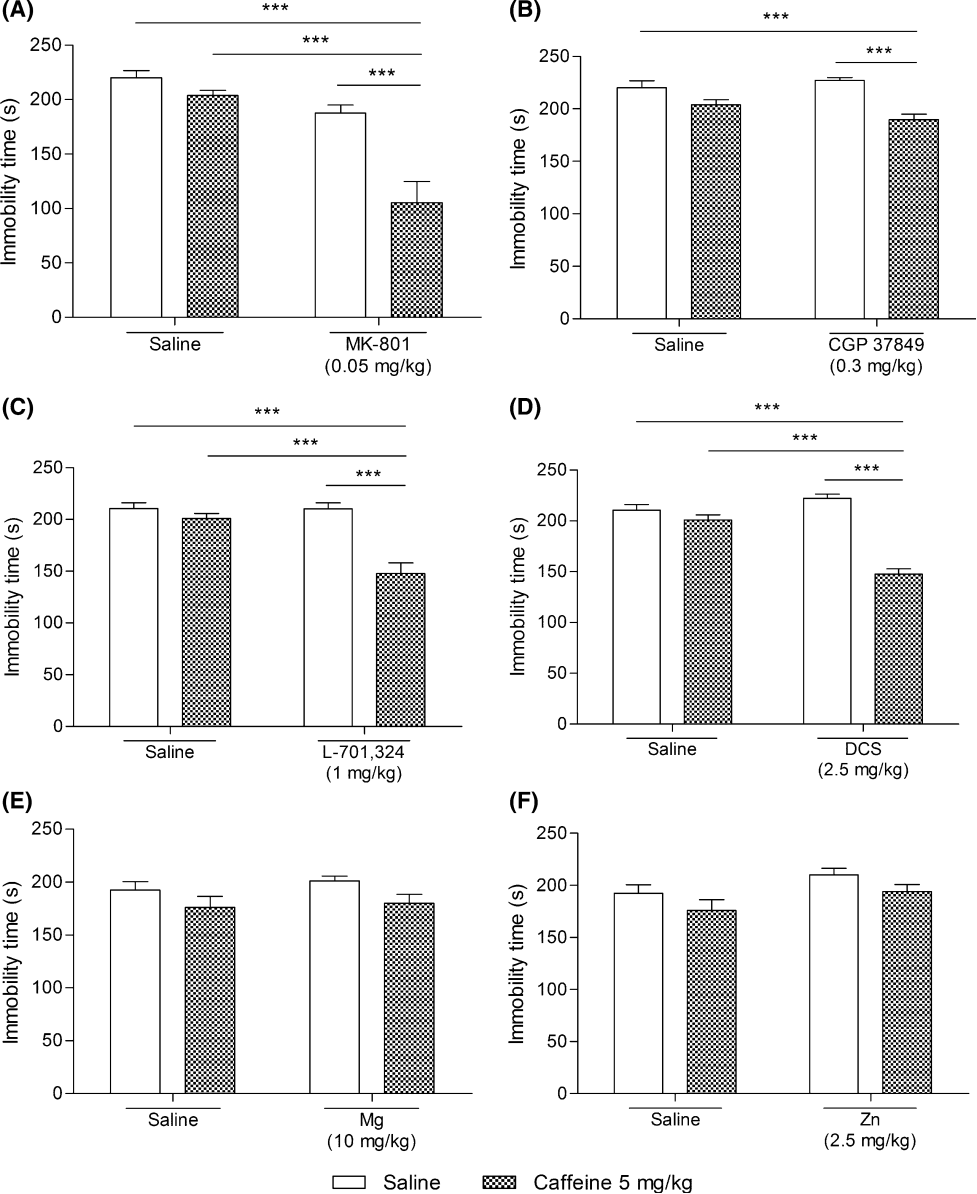
Effect of a joint administration of caffeine (5 mg/kg) and the NMDA receptor ligands: **a** MK-801 (0.05 mg/kg), **b** CGP 37849 (0.3 mg/kg), **c** L-701,324 (1 mg/kg), **d** d-cycloserine (DCS, 2.5 mg/kg), **e** magnesium hydroaspartate (10 mg/kg, Mg), and **f** zinc hydroaspartate (2.5 mg/kg, Zn) in the FST in mice. MK-801, CGP 37849, L-701,324, DCS and Zn were given 60 min before the experiment while caffeine and Mg were injected 40 and 30 min before the test, respectively. All agents were administered i.p. The control groups received saline. The values represent the mean + SEM (*n* = 9–10 mice per group). Two-way ANOVA for caffeine–treatment interactions: **a** *F*(1,35) = 9.55, *p* = 0.0039; **b** *F*(1,36) = 4.29, *p* = 0.0455; **c** *F*(1,36) = 14.38, *p* = 0.0005; **d** *F*(1,36) = 42.04, *p* < 0.0001; **e** *F*(1,36) = 0.08, *p* = 0.7823; **f** *F*(1,36) = 0.00, *p* = 0.9852; ***p < 0.001 (Bonferroni’s post hoc test)

**Fig. 3:**
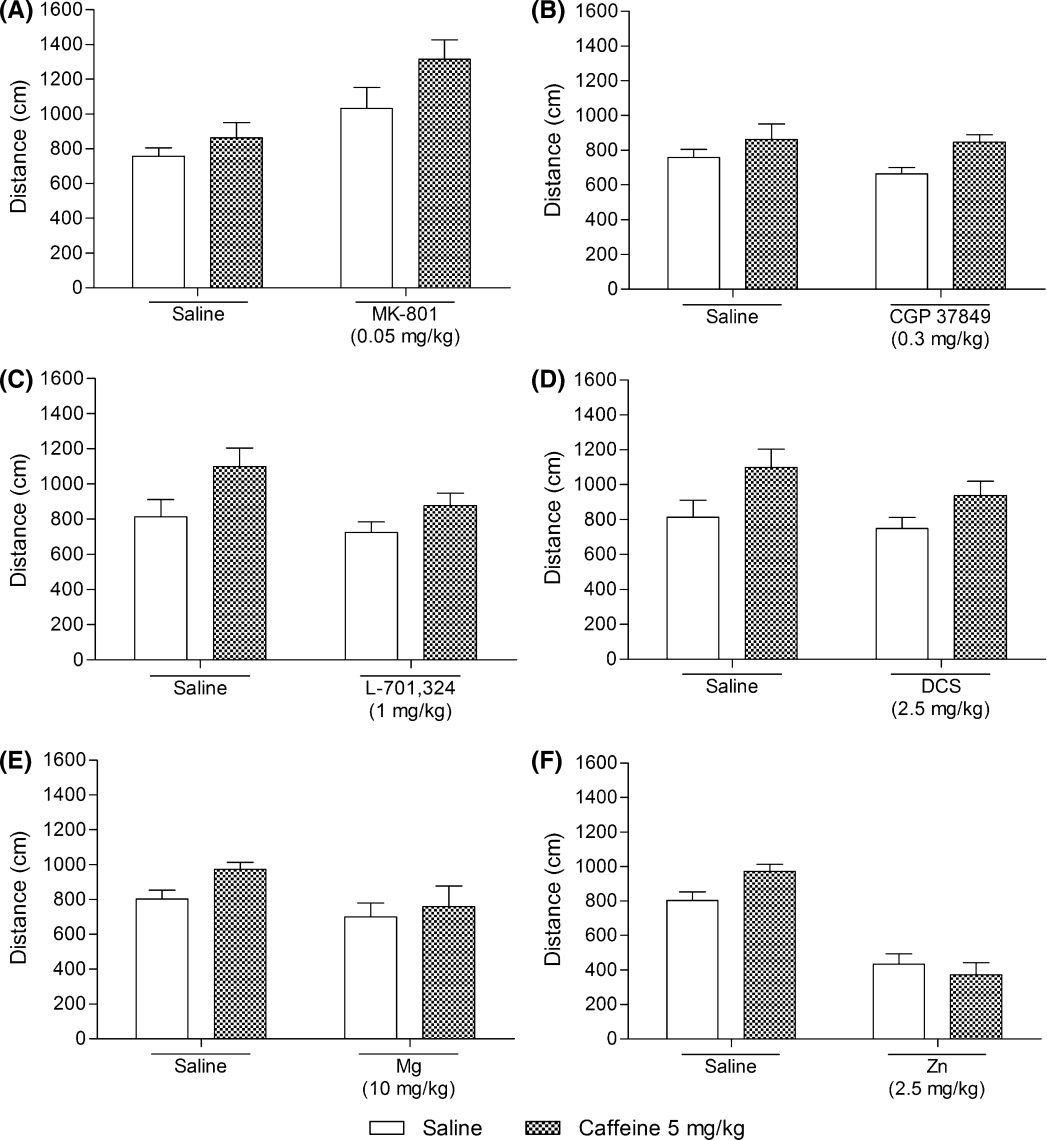
Effect of a joint administration of caffeine (5 mg/kg) and the NMDA receptor ligands: **a** MK-801 (0.05 mg/kg), **b** CGP 37849 (0.3 mg/kg), **c** L-701,324 (1 mg/kg), **d** d-cycloserine (DCS, 2.5 mg/kg), **e** magnesium hydroaspartate (10 mg/kg, Mg) and **f**, zinc hydroaspartate (2.5 mg/kg, Zn) on the spontaneous locomotor activity in mice. MK-801, CGP 37849, L-701,324, DCS, and Zn were given 60 min before the experiment while caffeine and Mg were injected 40 and 30 min before the test, respectively. All agents were administered i.p. The control groups received saline. The values represent the mean + SEM (*n* = 7–8 mice per group). Two-way ANOVA for caffeine–treatment interactions: **a** *F*(1,27) = 0.89, *p* = 0.3535; **b** *F*(1,27) = 0.45, *p* = 0.5074; **c** *F*(1,26) = 0.62, *p* = 0.4369; **d** *F*(1,26) = 0.30, *p* = 0.5891; **e** *F*(1,27) = 0.44, *p* = 0.5136; **f** *F*(1,26) = 3.96, *p* = 0.0572

The concurrent acute i.p. administration of caffeine (5 mg/kg) and MK-801 (0.05 mg/kg) induced an antidepressant-like effect in the FST in mice, which is illustrated in Fig. 2a. Two-way ANOVA revealed a significant caffeine–MK-801 treatment interaction with significant effects of both used agents. The post hoc analysis showed that co-administration of caffeine and MK-801 significantly (*p* < 0.001) shortened the total duration of immobility in mice, as compared to both the single-treated and vehicle-treated animals. Statistical analysis of the results obtained from the spontaneous locomotor activity indicated not significant caffeine–MK-801 treatment interaction with not quite significant effect of caffeine and significant effect of MK-801. Although neither caffeine nor MK-801 given separately as a single dose influenced the distance traveled by mice, the combination therapy increased the locomotor activity of animals in comparison to both saline- and caffeine-treated groups. The outcomes obtained in the spontaneous locomotor activity test are summarized in Fig. 3a.

### Effect of a joint administration of caffeine and CGP 37849 on the FST in mice

Caffeine and CGP 37849 administered at the doses of 5 and 0.3 mg/kg, respectively, did not induce a significant reduction in the immobility time of animals, but their co-administration shortened the total duration of immobility compared to caffeine and saline (*p* < 0.001) but not to CGP 37849 (*p* > 0.05). Accordingly, two-way ANOVA demonstrated that the caffeine–CGP 37849 treatment interaction was considered significant with a significant effect of caffeine and non-significant effect of CGP 37849 (Fig. 2b). The tested treatment combination did not affect the outcomes of the spontaneous locomotor activity (Fig. 3b). Two-way ANOVA demonstrated not significant effect of caffeine–CGP 37849 treatment interaction with significant effect of caffeine and not significant effect of CGP 37849.

### Effect of a joint administration of caffeine and L-701,324 on the FST in mice

Two-way ANOVA indicated a significant caffeine treatment × L-701,324 treatment interaction with significant effects of both caffeine (5 mg/kg) and L-701,324 (1 mg/kg). According to the post hoc analysis, the concomitant injection of *per se* inactive doses of caffeine and L-701,324 resulted in a considerable (*p* < 0.001) shortening of the total duration of immobility in mice compared with either drug alone as well as the vehicle-treated group (Fig. 2c). No modification in the animals’ spontaneous locomotor activity was recorded between the 2nd and the 6th minute of the experiment (Fig. 3c). Two-way ANOVA revealed not significant caffeine treatment × L-701,324 treatment interaction with significant effect of caffeine and not significant effect of L-701,324.

### Effect of a joint administration of caffeine and d-cycloserine on the FST in mice

Two-way ANOVA revealed a significant caffeine treatment × d-cycloserine treatment interaction with significant effects of both caffeine (5 mg/kg) and d-cycloserine (2.5 mg/kg). Relying on the post hoc outcomes, the combination of caffeine and d-cycloserine markedly reduced the immobility time of the animals, as compared to both single-treated groups (*p* < 0.001) and saline-treated mice (*p* < 0.001) (Fig. 2d). No significant difference was found in relation to the animals’ locomotion after administration of the tested substances (Fig. 3d). According to two-way ANOVA, caffeine–d-cycloserine treatment interaction was considered not significant with significant effect of caffeine and not significant effect of d-cycloserine.

### Effect of a joint administration of caffeine and magnesium hydroaspartate on the FST in mice

As seen in Fig. 2e, the joint administration of sub-effective doses of caffeine (5 mg/kg) and magnesium hydroaspartate (10 mg/kg) produced no antidepressant-like effect in the FST in mice. A non-significant caffeine treatment × magnesium hydroaspartate treatment interaction with a non-significant effect of magnesium hydroaspartate but a significant effect of caffeine were demonstrated by two-way ANOVA. Similarly, the administration of the tested agents did not alter the distance traveled by the animals (Fig. 3e). Two-way ANOVA showed not significant caffeine–magnesium hydroaspartate treatment interaction with no effect of either tested substance.

### Effect of a joint administration of caffeine and zinc hydroaspartate on the FST in mice

Caffeine (5 mg/kg) and zinc hydroaspartate (2.5 mg/kg) when given together did not exert any influence on the animals’ performance in the FST (Fig. 2f). Statistical analysis showed a non-significant caffeine treatment × zinc hydroaspartate treatment interaction with a non-significant effect of caffeine but a significant effect of zinc hydroaspartate. The outcomes of the spontaneous locomotor activity studies indicated not quite significant caffeine–zinc hydroaspartate treatment interaction with not significant effect of caffeine and significant effect of zinc hydroaspartate. However, both single injection of zinc hydroaspartate as well as its combination with caffeine considerably attenuated mice locomotion as compared to the saline-treated as well as saline-treated and caffeine-treated group, respectively. The results are presented in Fig. 3f.

## Discussion

The primary effects of caffeine include stimulation of the central-nervous system with reduction of fatigue, enhancement of mental performance, increase of alertness, and mood elevation (Winston et al. 2005). Although the pharmacological activity of caffeine is mediated via antagonism of the A_1_ and A_2_ receptors, caffeine at higher doses also inhibits phosphodiesterases, blocks the GABA_A_ receptor, and causes the mobilization of intracellular calcium (Daly and Fredholm 1998). Blockage of the adenosine receptor is known to reverse the inhibition of adrenaline release which leads to sympathetic stimulation. Moreover, through the antagonism of the A_2A_ receptors, caffeine indirectly influences the dopamine receptors. As was underlined by Ferre et al. (1992), an intact dopaminergic neurotransmission is required for the stimulatory effects of caffeine.

In the present study, we found that caffeine at a dose of 50 mg/kg reduced the immobility time in the FST to almost the same degree as an effective dose of imipramine, used as a positive control. In line with Kale and Addepalli (2014), a dose of 10 mg/kg (and 20 mg/kg) exerted the antidepressant-like activity, as well. Our observations confirm the reports on the relationship between caffeine consumption and the improved mood or reduction in depressive-like symptoms (Amendola and van Steensel 2014; Childs and de Wit 2008). Other adenosine A_2A_ receptor antagonists (e.g., SCH58261, ZM241385, KW6002) also reversed the signs of behavioral despair in the FST and the tail suspension test (TST) (El Yacoubi et al. 2003). Although several authors have reported that caffeine may increase the rodents’ locomotion in an inverted U-shaped manner (Finn and Holtzman 1987), none of the doses tested in our experiments influenced the locomotor activity of animals. Therefore, the recorded antidepressant-like effect of this methylxanthine was not due to its psychostimulant action. The same conclusions have been drawn by Pechlivanova et al. (2009). It is worth mentioning that the daily mean intake of caffeine reaches 1–2 mg/kg (i.e., 70–140 mg in a 70 kg individual), and this index seems to vary between countries and age groups. For example, for the Scandinavians, it even increases to 7 mg/kg/day which corresponds to about seven cups of coffee (Nehlig et al. 1992). However, one should remember that results from experiments based on the rodent models should not be directly extrapolated and applied to human conditions.

The non-effective dose of caffeine (5 mg/kg) given concurrently with also inactive doses of the NMDA receptor antagonists (i.e., MK-801, CGP 37849, L-701,324) or a partial agonist of a glycine recognition site (d-cycloserine) significantly reduced the immobility time of animals in the FST, suggesting a synergistic interaction between the tested agents. As for CGP 37849, L-701,324, and d-cycloserine, the observed outcomes were not influenced by the change in the overall spontaneous locomotor activity of the tested animals, since their respective combinations with caffeine did not increase the distance traveled by mice as compared with the control groups. Mice given MK-801 in combination with caffeine traveled a considerably longer distance than animals from the control groups, although neither MK-801 nor caffeine increased the animals’ locomotion by themselves. According to the literature data, MK-801 produces the motor-activating effects, though only after doses higher than the ones used in the present study (Dall’Igna et al. 2003). Similar to our results, co-administration of caffeine and ketamine (another antagonist of the NMDA receptor complex) significantly enhanced the ambulatory activity of mice (Uchihashi et al. 1992). Most probably, dopamine release was involved in the observed effects. Moreover, systematically administered caffeine potentiated the locomotor stimulation produced by MK-801 in mice (Kuribara et al. 1992). A pharmacological interaction between caffeine and MK-801 concerning the locomotor effect was also described by de Oliveira et al. (2005) and Dall’Igna et al. (2003). Subchronic and chronic exposure to caffeine resulted in the development of tolerance to MK-801-induced hyperlocomotion in mice. Contribution of the dopaminergic system to the increase of locomotor activity observed in our study after concomitant administration of caffeine and MK-801 cannot be ruled out. There are findings suggesting implication of the dopamine system in the locomotor stimulant effects of caffeine *per se*, whereas other studies indicate that caffeine enhances the locomotor activity effects produced by dopamine receptor agonists acting directly and indirectly (Garrett and Griffiths 1997). Fraser et al. (1997) revealed that the endogenous adenosine activity through the A_1_ receptors may be responsible, at least partially, for the locomotor stimulant properties of MK-801 recorded in the elevated plus maze test, while the motor-activating effects of acutely administered caffeine could have been mediated by a simultaneous blockage of the central adenosine A_1_ and A_2A_ receptors (Karcz-Kubicha et al. 2003).

The synergistic interaction between adenosine and glutamatergic systems detected in our study is not highly surprising, since such an interplay has been described in the literature (de Oliveira et al. 2005). Activation of the NMDA receptor induces adenosine release in the rat striatum and cortex (Craig and White 1993; Melani et al. 1999), while activation of the adenosine receptors reduces NMDA receptor-mediated effects (Sebastião and Ribeiro 2000). Theophylline (an antagonist of the adenosine receptor) given to haloperidol-treated animals enhanced the anticataleptic effects of NMDA receptor antagonists (Hauber and Munkle 1996). Moreover, NMDA receptor blockage counteracted the acute behavioral effects of caffeine withdrawal (Sukhotina et al. 2004), while adenosine receptor agonists counteracted the behavioral and neurophysiological changes caused by NMDA receptor antagonists in the animal models (Browne and Welch 1982; Popoli et al. 1997). Bespalov et al. (2006) demonstrated a synergistic interaction between NMDA receptor antagonists and caffeine. The observed effect was limited to the low dose of caffeine (i.e., 3 mg/kg), which was comparable to the one tested in our study. Such low caffeine doses act selectively through the adenosine receptors and produce stimulatory effects (Fredholm et al. 1999).

According to de Oliveira et al. (2005), an abrupt reduction of adenosine release may contribute to the locomotor and cognitive effects observed after NMDA receptor inhibition. It could explain the synergistic interaction between NMDA receptor antagonists and caffeine that was demonstrated in our study. However, the relationship between adenosinergic and glutamatergic systems is quite complicated, since co-administration of the sub-effective doses of adenosine with MK-801, ketamine, and zinc chloride also resulted in a synergistic effect in the FST, while administration of an active dose of A_1_ agonist (i.e., N^6^-cyclohexyladenosine) or adenosine prevented the binding of MK-801 to the NMDA receptor. Moreover, *N*-methyl-d-aspartate (NMDA receptor agonist) and d-serine (an endogenous agonist of the glycine site) prevented the anti-immobility effect induced by adenosine in the FST (Kaster et al. 2012). According to Kaster et al. (2012), the inhibition of the NMDA receptor mediated by the activation of the A_1_ receptors may underlie the antidepressant-like activity of adenosine.

Activation of adenosine A_1_ and A_2A_ receptors is also partially responsible for the antidepressant-like potential of zinc ions (Lobato et al. 2008), which could be an explanation for the lack of a synergistic interaction between caffeine and zinc hydroaspartate in the FST in the present study. Our observations are in accordance with the reports of Lobato et al. (2008), who showed that the anti-immobility effect of zinc chloride was reversed by pretreatment with the non-selective and selective antagonists of adenosine receptors (i.e., caffeine, DPCPX or ZM241385), given at sub-effective doses. On the other hand, both selective agonists of adenosine receptor (CHA, DPMA) and an adenosine transporter inhibitor (dipyridamole) enhanced the antidepressant-like effect of an inactive dose of zinc ions. Similar research should be performed in order to check if the same mechanisms are responsible for the lack of a synergistic interaction between caffeine and magnesium hydroaspartate.

It should be underlined that in view of several limitations of our study (small sample size, only one test performed, only one time point analyzed, no molecular analysis), the obtained results should be treated as the preliminary ones that need to be confirmed.

## Conclusion

The antidepressant-like potential of the NMDA receptor antagonists with the affinity to distinct binding sites (except for zinc and magnesium ions) was significantly augmented by the concomitant administration of caffeine. Therefore, the simultaneous blockage of the adenosine and NMDA receptors may offer an alternative target in the development of new pharmacological options for the treatment of depression.
